# A Phage Display Screening Derived Peptide with Affinity for the Adeninyl Moiety

**DOI:** 10.3390/bios4020137

**Published:** 2014-04-29

**Authors:** Louise Elmlund, Pernilla Söderberg, Subramanian Suriyanarayanan, Ian A. Nicholls

**Affiliations:** 1 Bioorganic & Biophysical Chemistry Laboratory, Linnæus University Centre for Biomaterials Chemistry, Linnæus University, SE-39182 Kalmar, Sweden; E-Mails: louise.elmlund@lnu.se (L.E.); pernilla.soderberg@lnu.se (P.S.); esusu@lnu.se (S.S.); 2 Department of Chemistry & Biomedical Sciences, Linnæus University, SE-39182 Kalmar, Sweden; 3 Department of Chemistry—BMC, Uppsala University, Box 576, SE-75123 Uppsala, Sweden

**Keywords:** phage display, piezoelectric sensor, QCM, adenine, peptide, molecular recognition

## Abstract

Phage display screening of a surface-immobilized adenine derivative led to the identification of a heptameric peptide with selectivity for adenine as demonstrated through quartz crystal microbalance (QCM) studies. The peptide demonstrated a concentration dependent affinity for an adeninyl moiety decorated surface (*K*_D_ of 968 ± 53.3 μM), which highlights the power of piezoelectric sensing in the study of weak interactions.

## 1. Introduction

Phage display technology [1,2,3] has proven a valuable tool for identifying peptidic motifs with affinities for target biomolecular structures. The concept of using phage display to develop biomolecular structures with function outside of a biological context, or even structures with functions not encountered in biology, is fascinating [4]. Nonetheless, this has received little attention to date with only a limited number of reports where the screening target is a material of non-biological nature [5,6,7,8], or where the screening is performed in an environment not generally encountered in biology [9]. In efforts towards engineering biomolecular structures capable of interaction with the surfaces of materials of non-biological origin, we undertook a proof-of-concept study using a cyclic heptapeptide phage library based upon the phagemid pG8H6. We have previously examined this phage with respect to its tolerance of organic solvents [10,11], where it was demonstrated that the phage could withstand significant concentrations of water miscible organic solvents, e.g., up to 99% acetonitrile. This surprising tolerance has been used by us for identifying peptides with affinity for alpha-chymotrypsin in media of low dielectricity [9]. The robustness of this phage is even demonstrated in its use for identifying peptides selective for synthetic polymers [7]. The adenine moiety was selected as the target for this study on account of its rigid structure, ready availability in a form amenable to immobilization [12] and its neutrality under the intended screening conditions in order to avoid indiscriminate ion-pairing interactions. In general, there are very few reported examples of using peptide phage display screening for small molecular targets, though the screening, again using the library employed in this study, for peptides selective the TNT is a noteworthy example [13].

## 2. Experimental Section

### 2.1. Chemicals & Materials

Chemicals and materials were obtained from commercial sources: adenine, chloroform, H_2_O_2_ (Merck, Darmstadt, Germany); ethylene carbonate, 1-ethyl-3-(3-dimethylaminopropyl)carbodiimide (EDC), NaOH, glass beads (106 micron and finer), NaIO_4_, 4-(dimethylamino)pyridine (DMAP), 11-MUA, ethanolamine (Sigma-Aldrich, St. Louis, MO, USA); *N,N’*-dimethylformamide (DMF) (BDH, through VWR International, Briare, France); allylcholorodimethylsilane (ACDMS), *N,N'*-dicyclohexylcarbodiimide (DCC), KMnO_4_ (Fluka, Buchs, Switzerland); H_2_SO_4_ (Riedel-de-Haën, through Sigma-Aldrich, Buchs, Germany); acetone (VWR International, Briare, France). All solvents and reagents were of analytical grade. Ph.D.^TM^-C7C Phage Display peptide library kit and *Escherichia Coli* (*E. coli*) ER2738 were obtained from New England BioLabs (Ipswich, MA, USA). Unless otherwise stated, the term *buffer* refers to 25 mM sodium phosphate, pH 7.8. Water was purified using a Milli-Q purified water (Millipore AB, Billerica, MA, USA).

### 2.2. Synthesis Protocols

#### 2.2.1. Synthesis of 9-(2'-hydroxyethyl)adenine

**Figure 1:**
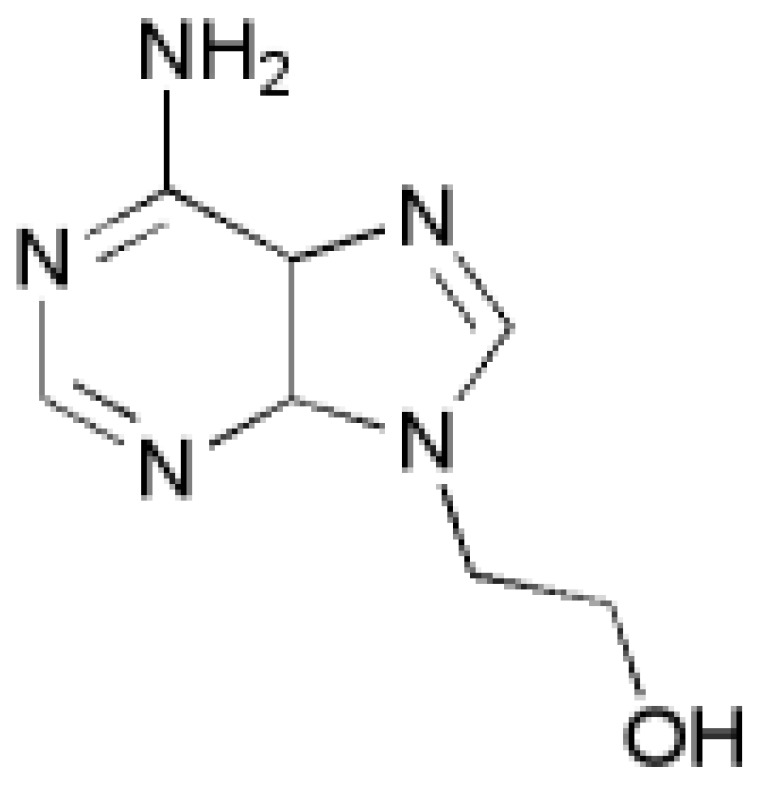
Structure of 9-(2′-hydroxyethyl)adenine.

Adenine (4.96 g, 36.72 mmol), ethylene carbonate (3.31 g, 38.41 mmol) and NaOH (one pellet) were mixed in DMF (150 mL) and heated at reflux for 2 h. The solvent was removed by evaporation *in vacuo*. Recrystallization from ethanol furnished the desired product (Figure 1) as white needles (1.77 g, 27%, mp 238–241 °C, Lit. 238–239 [14]) after drying under vacuum. ^1^H- and ^13^C-NMR spectral properties were in agreement with those previously reported [12]: δ_H_ (500 MHz; d_6_-DMSO) 8.13 (1 H, s, ArH), 8.07 (1 H, s, ArH), 7.18 (2 H, s, NH_2_), 5.02 (1H, t, *J* = 5.3 Hz, OH), 4.18 (2 H, t, *J* = 5.6 Hz, CH_2_), 3.73 (2 H, q, *J* = 5.4 Hz, CH_2_); δ_C_ (126 MHz; D_6_-DMSO) 155.94, 152.29, 149.57, 141.39, 118.73, 59.30, 45.76. 

#### 2.2.2. Derivatization of Glass Beads

*Silanization*—In a typical preparation, glass beads (10 g) were washed for 10 min at 80 °C water bath in “piranha” solution (H_2_O_2_ (30% v/v)—conc. H_2_SO_4_, 1:3, 20 mL), cooled to RT and washed with water (5 × 1 min) followed repeated washing with dry acetone (20 mL, 5 × 1 min) and finally dry chloroform (20 mL, 5 × 1 min), prior to vacuum filtration. *Caution:* “*Piranha*”* solution must be handled with extreme care since it is a hazardous oxidizing agent and reacts violently with most organic materials!* The beads were then placed in a solution of allylcholorodimethylsilane in chloroform (5%, v/v), and gently stirred in this solution at room temperature (16 h). The beads were filtered and washed with chloroform (5 × 20 mL) before being stored in a desiccator.

*Oxidation*—In a typical study adapted from that of von Rudloff [15], beads (25.0 g, two batches) were put in a round bottle flask and acetone (20 mL) was added. While stirring, a solution of KMnO_4_ (304 mg, 1.90 mmol) and NaIO_4_ (10 g, 46.8 mmol) mixed in water (100 mL) was added dropwise, reaction left overnight. Aqueous HCl (40 mL, 10% v/v) was added and the mixture was stirred for 1 h. The beads were washed in 3× water and thereafter stirred for 80 min in 2 × 50 mL ethanol (99.7%) to eliminate manganate precipitate. The solved precipitate was seen again after wash in water but disappeared when the beads was treated with ethanol (99.7%) under gentle heating. The beads were filtered through a filter paper and washed with water before they were air-dried, resulting in 10.8 g. 

*Esterification*: Oxidized beads (10.6 g), 9-(2′-hydroxyethyl)adenine (1.12 g, 6.24 mmol), *N,N*′-dicyclohexylcarbodiimide (DCC) (1.34 g, 6.5 mmol) and 4-(dimethylamino)pyridine (DMAP) (0.786 g, 6.43 mmol) were heated at reflux in dry DMF (50 mL) for 4 h. The beads were collected by filtration, rinsed with water (150 mL) and air dried overnight. To provide further confirmation of the outcome of the chemistry on the glass beads, pieces of silicon wafer (Aldrich, 10 × 10 mm, 0.5 mm thick, single side polish, 28.1 g/mol) were derivatized using the same procedure.

### 2.3. Biopanning with the Ph.D.TM-C7C Library

The phage library (10 μL, 10^11^ phage) was mixed with buffer (990 μL), to a final volume of 1000 μL, 20 μL was withdrawn for titration. The phage solution was immediately added to the adenine immobilized glass beads (50 mg) followed by incubation (30 min) on a rocking table and brief centrifugation to sediment the beads. An aliquot (20 μL) was removed for titration before removal of the supernatant. The beads were washed three times (3 min) with 1 mL aliquots of buffer on a rocking table followed by a short start-stop spin centrifugation and removal of supernatant. Elution of bound phages was performed on a rocking table during 30 min using 1 mL adenine in buffer (10^−4^ M, biopanning round one and two) followed by a short start-stop spin centrifugation and a volume of 20 μL was withdrawn for titration. The eluted phage were amplified and used in a new round of biopanning. In the third round of biopanning, elution was performed with both 10^−6^ M and 10^−4^ M adenine in buffer. From this last biopanning, 20 individual clones were isolated from each elution, in total 40 clones, and were amplified and sequenced. Biopanning rounds two and three employed an input of 10^12^ phage.

#### 2.3.1. Strains and Media

*E. coli* ER2738 was grown overnight on LA plates (LB medium with agar, 15% (w/v)) containing tetracycline (20 μg·mL^−1^) at 37 °C and then stored at 4 °C. Before amplification, titration and DNA purification, colonies were picked and grown in LB medium (peptone (1%), yeast extract (0.5%), NaCl (0.5%) (w/v) in H_2_O) at 37 °C with shaking until log growth phase was reached. 

#### 2.3.2. Amplification of Eluted Phages and Single Phage Clones

From a phage eluate, 10 aliquots of 100 μL were separately grown overnight in 10 mL LB infecting *E. coli* ER2738 (100 μL in log phase) with shaking at 37 °C. Volumes (2 × 1.5 mL) from each solution were centrifuged at 14,000 rpm, 4 °C for 5 min. Supernatant (1.2 mL) was removed from each tube and added to 180 μL precipitation medium (PEG6000 (21%, w/v), NaCl (24.5%, w/v) in H_2_O). Samples were vortexed and placed on ice for 2 h, then centrifuged at 14,000 rpm, 4 °C for 40 min. The supernatants were removed and the 20 phage pellets were resuspended and pooled in a total volume of 200 μL buffer. The resultant sample constituted a new phage lysate. Single phage clones from the final round of biopanning, in total 40, were collected using a sterile inoculation loop, were also amplified in LB overnight and further purified as described above.

#### 2.3.3. Titration

From a phage dilution series, 100 μL aliquots were added to *E. coli* ER2738 (200 μL, in log phase) and incubated at 20 °C for 6 min. Aliquots (100 μL) were then added to 3 mL top agar (peptone (1%), NaCl (0.8%), agar (0.40%) (w/v) in H_2_O), vortexed and poured onto LA plates containing Xgal (40 μg·mL^−1^) and IPTG (50 μg·mL^−1^) followed by incubation overnight at 37 °C. Bacterial colonies, corresponding to the number of infectious phages, were counted the following day. The quantification of phages using “*efficiency of plating*” was employed rather than a UV determination, in order to have an estimate of the number of viable phages. 

#### 2.3.4. DNA Purification of Single Phage Clones

An aliquot from each phage clone (5 μL) was added to 11 mL LB containing 100 μL *E. coli* ER2738 in log phase and incubated overnight on a shaker at 37 °C. Each culture was divided into seven aliquots of 1.4 mL each in eppendorf tubes, and centrifuged at 14,000 rpm, 20 °C for 5 min. The supernatants were mixed with 400 μL precipitation medium (PEG8000 (20%), NaCl (14.6%) (w/v) in H_2_O) and incubated at 20 °C for 10 min, then centrifuged for 10 min as above. Pellets were thoroughly suspended in 100 μL iodide buffer (10 mM Tris-HCl, pH 8.0) followed by addition of 250 μL ethanol (99.7%), vortexing and incubation at 20 °C for 10 min. The suspensions were centrifuged for 10 min as above and the resulting pellets were washed with 1 mL ethanol (70%) and dried under vacuum. The seven DNA pellets from each clone were suspended and pooled in a total volume of 40 μL H_2_O. Further DNA purification by re-precipitation was done by addition of 4 μL sodium acetate (3 M, pH 5.2) and 110 μL ethanol (99.7%) to each 40 μL solution, followed by incubation at −20 °C for 1 h. The suspensions were then centrifuged at 13,200 rpm, 4 °C for 30 min, supernatant was removed and the resulting pellets were dried under vacuum. Pellets were dissolved in 50 μL H_2_O. DNA purity was analyzed on a NanoDrop spectrophotometer (Thermo, UK) and solutions containing 2 μg DNA were dried under vacuum before being sequenced (MWG, Germany). Two versions of these two peptide sequences were synthesized, one with *N*-acetylation and amidation, and the second with just amidation (Federation Bioscience, Australia).

#### 2.3.5. Adenine Viability Assay

Prior to all tests, a stock solution of adenine (1.85 mM) was prepared by mixing adenine and buffer followed by dilution to desired concentrations (10^−3^ M, 10^−4^ M, 10^−5^ M, 10^−6^ M, 10^−7^ M or 10^−8^ M). A zero sample, only buffer, was also prepared. The phage library (10 μL, 10^11^ phage) was diluted in 1490 μL buffer and aliquoted into eppendorf tubes with 21 μL in each. The aliquoted phage (2.8 × 10^9^ pfu/tube) were treated with all the different adenine concentrations in a total volume of 100 μL for 30 min at 20 °C. Titration was performed as described above. The assay was performed in triplicate of each sample. This procedure was used in order to ensure viability of the phage library in presence of adenine.

### 2.4. Preparation of Adenine Coated Quartz Crystal Resonators

Quartz Crystal Microbalance resonators (QCM surfaces) sputtered with gold on each side (AT cut, 10 MHz fundamental frequency, 7 mm in diameter), were obtained from Attana AB (Stockholm, Sweden). First, the resonators were cleaned in “piranha” solution (1:3 v/v mixture of 30% H_2_O_2_/conc. H_2_SO_4_) for 2 min followed by generously rinsing in water and finally once in ethanol (99.7%). The QCM surfaces were immersed in a 5 mM 11-mercaptoundecanoic acid solution in ethanol (99.75%) on a rocking table over night. The surfaces were then rinsed once in ethanol and dried under a stream of dry N_2_ (g). 9-(2′-hydroxyethyl)adenine (10 mM), DMAP (15 mM) and 1-ethyl-3-(3-dimethylaminopropyl)carbodiimide (EDC, 75 mM) were solved in dry DMF (10 mL) and the QCM resonators were immersed in the solution over night, on a rocking table. Finally, the resonators were rinsed once in chloroform and dried in N_2_ (g), and were stored dark at 4 °C in eppendorf tubes until use.

### 2.5. Characterization of Adenine Coated Surfaces

#### 2.5.1. RAIR Spectroscopy

RAIR spectra of the adenine modified Au/quartz resonator surfaces and silicon wafer (used for standardizing the chemistry) were recorded on a Bruker Hyperion 3000 IR microscope attached to a Tensor 27 IR spectrometer and computer-controlled sample stage. The infrared beam was double surface reflected at angles of 52° and 83° to the surface normal using a grazing angle objective. The spectra were obtained from acquisitions of 1000 interferograms collected using a single element mercury-cadmium-telluride (MCT) detector with a resolution of 4 cm^−1^. During the measurements, inert atmosphere was maintained within the sample chamber by purging with nitrogen gas at positive pressure. A three-term Blackmann–Harris apodization function was applied to the interferograms, prior to the Fourier transformation. Piranha cleaned beads, silicon wafers and/or Au/quartz resonators were used as reference surfaces. The spectra of the adenine modified glass beads were recorded on a Nicolet Avatar instrument. 

**Figure 2:**
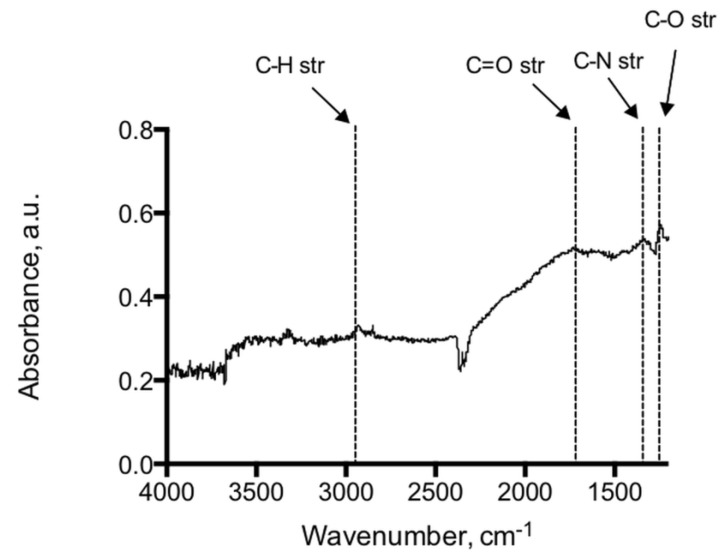
IR-spectrum of the adenine coated glass beads showed peaks at 1246 cm^−1^, 1334 cm^−1^, 1728 cm^−1^ representing C–O, C–N and C=O functional moieties of the immobilized adenine. Peaks at 2932 cm^−1^ and 2851 cm^−1^ are indicative of the methylene groups of the alkyl backbone.

**Figure 3:**
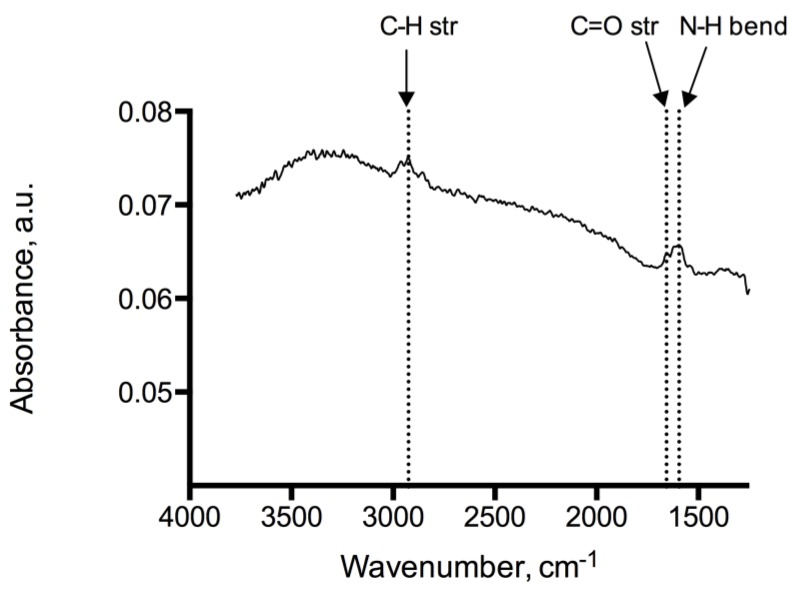
IR-spectrum of the adenine coated silicon wafers showed peaks at 1595–1657 cm^−1^ and 2858–2925 cm^−1^ typical for C=O, N–H and C–H stretching modes.

**Figure 4:**
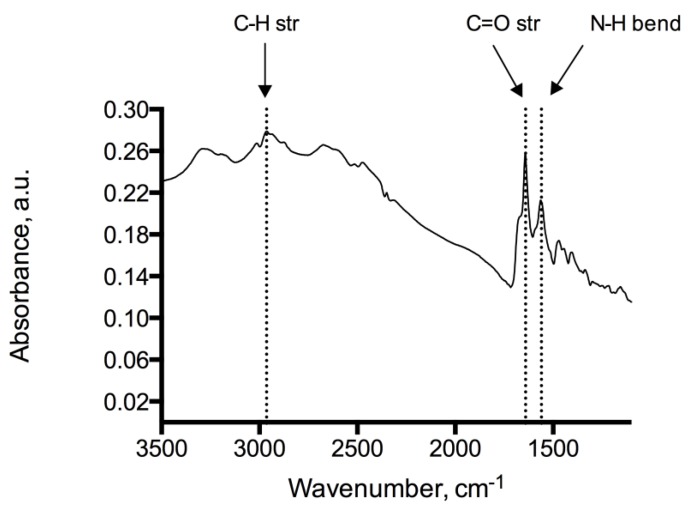
IR-spectrum of the adenine coated Au/quartz surface showing N–H (1560 cm^−1^), C=O (1641 cm^−1^) and C–H (2964 cm^−1^) absorptions.

Analysis of the adenine coated glass beads show peaks at 1246 cm^−1^, 1334 cm^−1^, 1728 cm^−1^ representing C–O, C–N and C=O functional moieties of the immobilized adenine, see Figure 2. Peaks at 2932 cm^−1^ and 2851 cm^−1^ indicate methylene groups of the alkyl backbone. RAIR measurements of the adenine derivatized silicon wafer reveal peaks in 1595–1657 cm^−1^ and 2858–2925 cm^−1^ typically for C=O, N–H and C–H stretching modes, which provides evidence of the successful immobilization on glass beads and silicon wafers (Figure 3). In addition, the RAIR spectra trace of the adenine immobilized Au/quartz surface clearly represents C=O, N–H and C–H moieties of adenine, see Figure 4. 

#### 2.5.2. XPS

X-ray Photoelectron Spectroscopy (XPS) measurements were performed on a Physical Electronics Quantum 2000 Scanning ESCA Microprobe equipped with a monochromatic AlKα X-ray source (hv = 1486.6 eV). The pass energy was 117.40 eV for the survey spectra that was collected in the range 0 to 1100 eV. The Au4f and N1s core level spectra were recorded with 23.50 eV pass energy and 0.1 eV increment. The pressure in the sample chamber was kept constant at 5 × 10^−9^ mbar. The spectral background was corrected based on Shirley method of data processing and the spectral bands were deconvoluted for different nitrogen atoms.

Binding energy profiles of the gold surface recorded before and after adenine immobilization reveals essential peaks at 85, 286, 401 and 533.5 eV, respectively, for Au 4f7, C 1s, N 1s and O 1s elements. The peak for nitrogen at 401 eV is the benchmark confirming adenine adsorption that was absent on the binding energy profile for bare gold surface. Upon deconvolution this peak can be resolved into three corresponding the three different nitrogen atom viz., NH_2_ (400.05 eV), –NH– (403.1 eV) and =N– (401.4 eV) present in the adenine molecule. 

### 2.6. QCM Measurements

The selectivity of the peptides to adenine coated QCM chips was studied using an Attana Cell 200 instrument (Attana AB, Stockholm, Sweden) under FIA conditions. The biosensor contains a dual channel system with continuous flow and temperature control suitable for real time binding studies. The QCM surface was placed into chip holder (from manufacturer) before inserted into instrument. A continuous flow of buffer (20 μL/min) was passed over the surface until stabilization of baseline was reached. Two injections of ethanolamine (1 M) were passed over the surface to block unreacted carboxyl groups, followed by passage of buffer to reach stabilization of baseline. The binding study can be summarized as following: 35 μL analyte (peptide solved in buffer) was injected to QCM surface followed by dissociation (5 min) under continuous flow of buffer. To regenerate the surface, 35 μL of 25 mM Na_2_HPO_4_/NaH_2_PO_4_ (pH 10) was injected followed by stabilization of baseline (10 min) with buffer. All measurements were performed at 22 °C. Data were collected and evaluated using software from manufacturer (Attester Evaluation, Attana AB). A carboxyl surface was used as reference since adenine was coated on a carboxyl surface. 

## 3. Results and Discussion

### 3.1. Phage Display Screening

Three rounds of biopanning were performed using the cyclic heptamer presenting library (Ph.D.^TM^-C7C) with 30 min incubations. As the target, the adeninyl moiety, is limited in size and functionality, a cyclic peptide library was selected for use, the constraints imposed by the cyclization being assumed to present a more defined (less flexible) peptide structure. After each round the beads were washed with buffer then phage were eluted by incubation (30 min) in 10^−4^ M adenine solution. It is important to note that the possible influence of adenine on phage viability was also examined (over the range 10^−8^–10^−3^ M), with no adverse effect being observed. The phage were subsequently amplified in *E. coli* ER2738 on top agar coated LA plates containing Xgal [16], before titration and use in the subsequent rounds (10^12^ phage in each of the last two rounds, performed as above). Quantification of phage was performed using “*efficiency of plating*” rather than a UV-based determination, in order to obtain an estimate of the number of viable phage. Forty clones were collected from the final round. Each clone was subsequently amplified, and the DNA isolated and purified by re-precipitation. Sequencing revealed the insert to be present in three clones from the final elution, respectively.

Two of the peptides were selected for establishing possible affinity for adenine. Both were synthesized with a series of flanking residues that included the cysteines necessary for forming the disulphide bridge (AC-xxxxxxx-CGG) that restrains the variable domain in the phage library. Two versions of each peptide were prepared, the first with the *N*- and *C*-termini acetylated and amidated, respectively, and the second with only amidation, Table 1. These modifications of the termini were used so that the peptides would better resemble the environment of the selected sequences present in the variable domain of pIII (HSAC-xxxxxxx-CGG) during biopanning. 

**Table 1:** Sequences for peptides derived from screening and synthesis.

	Aa sequence
pIII sequence A	--HSAC***RADYYAS***CGG--
Synthesized peptide AI	Ac-AC***RADYYAS***CGG-NH_2_
Synthesized peptide AII	AC***RADYYAS***CGG-NH_2_
	
pIII sequence B	--HSAC***HASSLPT***CGG--
Synthesized peptide BI	Ac-AC***HASSLPT***CGG-NH_2_
Synthesized peptide BII	AC***HASSLPT***CGG-NH_2_

### 3.2. Peptide-Adeninyl Moiety Decorated Surface Recognition Studies

Peptide-adenine interactions were then investigated using a series of quartz crystal microbalance (QCM) studies under flow injection analysis (FIA) conditions. Gold sputter-coated quartz resonators were first treated with 11-mercaptoundecanoic acid (11-MUA) before the coupling of 9-(2′-hydroxyethyl)adenine using 1-ethyl-3-(3-dimethylaminopropyl)carbodiimide in the presence of DMAP in dry DMF. A combination of RAIR and X-ray photoelectron (XPS) spectroscopies confirmed the presence of the anticipated functionalities on the resonator surfaces. The 11-MUA coated resonators were also used for control studies to assess the extent of peptide binding not directed specifically to the adeninyl moiety. 

**Figure 5:**
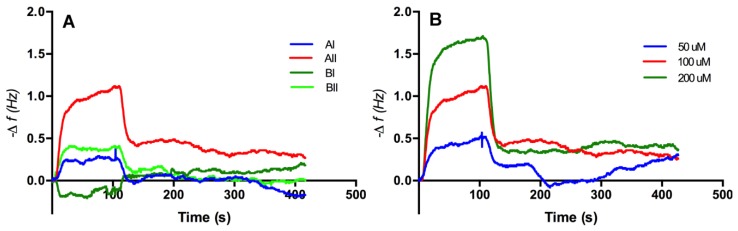
Representative frequency *versus* time response curves for injections of 100 μM of each synthesized peptides (**A**) and peptide AII interaction with the adenine surface in different concentrations (**B**). Volume injected of the peptide was 35 μL at a flow rate of 20 μL/min. Buffer (pH 7. 8) was used as a carrier solution.

**Figure 6:**
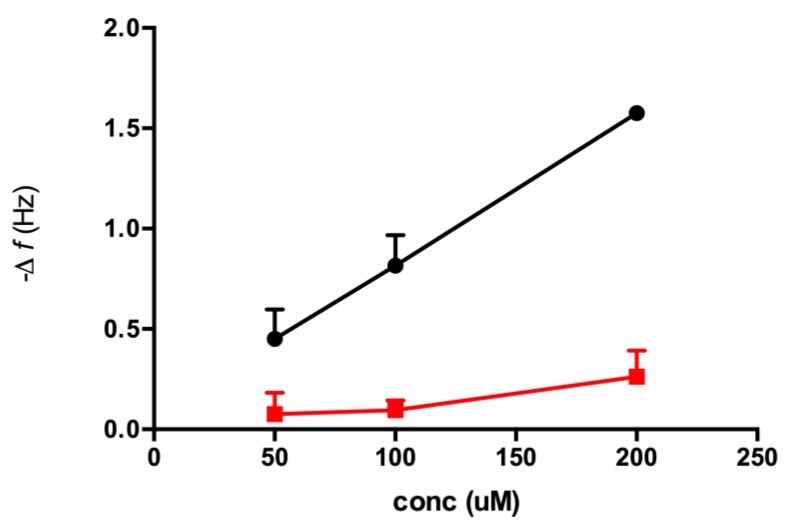
Maximum resonant frequency shift for Peptide AII interaction with the adenine surface (black) and carboxyl surface (red), respectively. Data from three injections of each concentration on respective resonators. Error bars represent the SEM.

Sensograms obtained from the injection of solutions of the peptides (50–200 μM, 35 μL) in buffer (NaH_2_PO_4_/Na_2_HPO_4_, 25 mM, pH 7.8) with a flow rate of 20 μL/min over the adenine decorated and control sensor surfaces revealed that only one of the peptides, peptide AII, produced a reproducible change in sensor resonant frequency commensurate with an increase in mass at the sensor surface, Figure 5A. Furthermore, and importantly, this change was demonstrated to be concentration dependent, Figure 5B. The fast apparent off-rate and fast recovery times reflect the relatively low affinity of the peptide for the surface, though at the same time highlighting the sensitivity of the technique. To establish the influence of the adeninyl moiety on this interaction, control experiments were performed using 11-MUA coated resonators, Figure 6. In all studies, ethanolamine was used to block residual surface carboxyl functionalities. Significantly, the adenine-derivatized surfaces had an affinity for peptide AII that was an order of magnitude greater than that of the control surfaces that lacked the immobilized adenine moiety. A closer examination of the affinity of peptide AII (P) for the adeninyl moiety (A) was quantified, using the method of Skladal [17], by determining the apparent stability constant (*K*_s_), of the non-covalently bound [A–P] biocomplex [18] (Equation (1)). Formation of the affinity complex between adenine ligand and peptide is characterized by the corresponding association (*k*_a_) and dissociation (*k*_d_) rate constants, and can be derived with the kinetic equation (Equation (2)) using the measured frequency *f* and concentration of the peptide *c* as reported elsewhere [19,20,21,22,23,24].

[A] + [P] ⇔ [A–P]
(1)

*f* = *f*_eq_ [1 − exp(−*k*_obs_ t)] (2)

where *k*_obs_ = k_a_*c*_T_ + *k*_d_ and *c*_T_ is the concentration of analyte.

**Figure 7:**
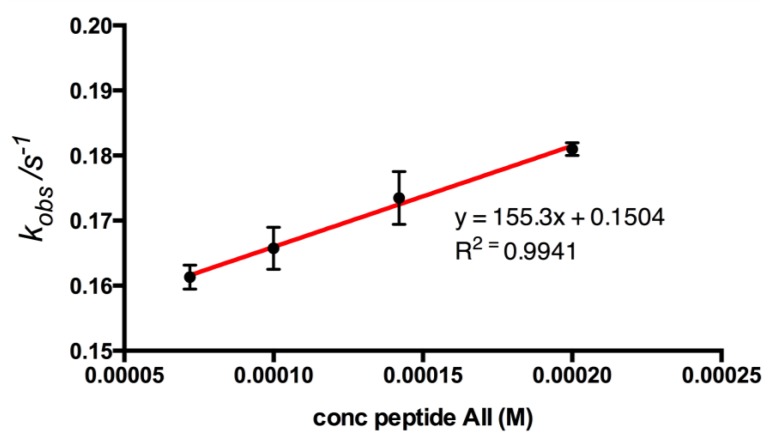
Plot of *k*_obs_ constants against corresponding concentrations of peptide AII to adenine surface. The *k*_obs_ values were calculated using non-linear curve fitting (Origin software, OriginLab Corporation, USA), from the association part of QCM binding curves (n = 3). A linear regression curve gives information of association and dissociation rates for peptide AII. Association rate constant (*k_a_*) was calculated to 155 M^−1^·s^−1^ and dissociation rate constant (*k_d_*) to 0.150 s^−1^ for the peptide, corresponding to an apparent *K*_D_ = 968 ± 53.3 μM.

The apparent rate constants *k*_obs_ can be deduced by fitting the initial parts of the FIA binding curves (Figure 4) for the different bupivacaine concentrations to Equation (1) [17,25,26,27]. The determined *k*_obs_ values varied linearly with the concentration of the analyte, Figure 7. Apparent stability constant (*K*_s_ = *k*_a_/*k*_d_) [17] can be calculated from the ratio of the slope (*k*_a_) and intercept (*k*_d_) of this plot.

The affinity of peptide AII for the adenine-decorated surfaces revealed a *K*_D_ value of 968 ± 53.3 μM. The magnitude of the observed binding reflected the limited size (135 g/mol) functionality of the adeninyl moiety. The greater affinity of peptide AII for adenine relative to that of peptide AI suggests that functionality with positive charge character in the vicinity of the *N*-terminus of this sequence may contribute to peptide-adenine affinity. However the lack of affinity observed for peptide BII, also presenting a free *N*-terminus indicated that peptide AII’s affinity is also steered through a sequence-specific interaction, Table 1. Interestingly, the adeninyl moiety-decorated QCM resonators demonstrated comparable performance after a one year period of storage in the dry state.

The selected sequence present in peptide A and its synthesized derivatives include a combination of acidic, basic, neutral and hydrophobic residues, which is in contrast to the corresponding sequence of peptide B as it is devoid of charged residues at this pH. In particular, the sequence of peptide A derived from the biopanning was zwitterionic with charged arginine and aspartic acid residues. We suggest that this peptide’s affinity for the adeninyl moiety may be in part derived from Coulombic interactions in combination with weaker contributions from the other residues, though further structure-activity studies using alternative peptides are required to confirm this hypothesis. Furthermore, the presence of a positively charged *N*-terminus in the vicinity of these charged residues appears to enhance the affinity. 

## 4. Conclusions

Collectively, the identification of the cyclic heptameric peptide AII constitutes, to the best of our knowledge, the first example of the use of phage display screening for identifying structures selective for surface immobilized small organic structures. As the recognition of very small organic structures by biomacromolecules is generally limited, this study demonstrates the potential of phage display for the generation of peptidic structures for small targets, and highlights the power of piezoelectric sensing in the study of weak interactions.
